# Hsa-miR-134 suppresses non-small cell lung cancer (NSCLC) development through down-regulation of CCND1

**DOI:** 10.18632/oncotarget.8482

**Published:** 2016-06-14

**Authors:** Cheng-Cao Sun, Shu-Jun Li, De-Jia Li

**Affiliations:** ^1^ Department of Occupational and Environmental Health, School of Public Health, Wuhan University, Wuhan, P. R. China; ^2^ Wuhan Hospital for The Prevention and Treatment of Occupational Diseases, Wuhan, P. R. China

**Keywords:** hsa-miRNA-134 (miR-134), cyclin D1, non-small cell lung cancer (NSCLC), proliferation, apoptosis

## Abstract

Hsa-miRNA-134 (miR-134) has recently been discovered to have anticancer efficacy in different organs. However, the role of miR-134 on non-small cell lung cancer (NSCLC) is still ambiguous. In this study, we investigated the role of miR-134 on the development of NSCLC. The results indicated that miR-134 was significantly down-regulated in primary tumor tissues and very low levels were found in NSCLC cell lines. Ectopic expression of miR-134 in NSCLC cell lines significantly suppressed cell growth as evidenced by cell viability assay, colony formation assay and BrdU staining, through inhibition of cyclin D1, cyclin D2, CDK4 and up-regulation of p57(Kip2) and p21(Waf1/Cip1). In addition, miR-134 induced apoptosis, as indicated by concomitantly with up-regulation of key apoptosis protein cleaved caspase-3, and down-regulation of anti-apoptosis protein Bcl2. Moreover, miR-134 inhibited cellular migration and invasiveness through inhibition of matrix metalloproteinases (MMP)-7 and MMP-9. Further, oncogene *CCND1* was revealed to be a putative target of miR-134, which was inversely correlated with miR-134 expression in NSCLC. Taken together, our results demonstrated that miR-134 played a pivotal role on NSCLC through inhibiting cell proliferation, migration, invasion, and promoting apoptosis by targeting oncogenic *CCND1*.

## INTRODUCTION

MicroRNAs (miRNAs) are a class of small, highly conserved, and non-coding RNAs that directly target genes' 3′-untranslated regions (3′-UTRs) by binding to some sequence-specific sites, resulting in deceased expression of these genes [1–3]. Numerous of publications have confirmed that dysregulation of miRNAs play an important role in various types of cancers [4–9]. Selective miRNA expression contributes to tumor proliferation, apoptosis, senescence, cell identity, stem cell maintenance and metastasis [4–16]. While there are still numerous of unknown details about the role of miRNAs on human cancers that still need to be investigated [17].

Lung cancer is one of the leading causes of cancer mortality around the world. There are estimated to be 1.80 million new cases in 2012, killing about 1.59 million people per year globally, extrapolating from a 2012 International Agency for Research on Cancer (IARC) risk assessment [18, 19], and this trend is expected to continue until 2030. Generally, approximately 85% of lung cancers are classified histopathologically as non-small cell lung carcinomas (NSCLC). Platinum-based chemotherapy was used to treat NSCLC, and got some [18], but the 5-year overall survival (OS) rate of it was just 16% for all stages [19, 20]. The mechanisms of lung cancer are attributed to silencing of tumor suppressor genes, dysregulation of proto-oncogenes, and an up-regulation of genes that promote cell growth and transformation and ultimately tumor development [19–23].

MiR-134, a recognized tumor-suppressing miRNA, has been shown to be down-regulated in a variety of diseases [24] including cancers, such as melanoma [25], breast cancer [26, 27], ovarian cancer [28], renal cell carcinoma [29], endometrial cancer [30], embryonal carcinoma [31], hepatocellular carcinoma [32, 33], head and neck carcinoma [34] and glioblastoma [35]. It is also reported that miR-134 regulates small cell lung cancer (SCLC) cell H69 growth and apoptosis by suppressing the ERK1/2 signaling pathway and directly targeting WWOX gene [36]. MiR-134 could also make sense in regulation of cell growth, metastasis, and apoptosis involving lung septation [37]. In addition, miR-134 suppresses epithelial to mesenchymal transition (EMT) by targeting oncogenic FOXM1 gene in NSCLC cells [38]. These results suggest tumor-suppressive functions of miR-134 on lung cancer but up to now this suggestion has not been rigorously tested.

The goal for our current study is to investigate the biological functions of miR-134 in NSCLC and to explore the underlying mechanisms of action. We find that miR-134 directly targets 3′-UTR of human CCND1 mRNA, which is up-regulated in many cancers, including lung cancer. Cyclin D1 is encoded by *CCND1* gene, and is an important oncogene that shown strong power of oncogenicity, by promotion of cell growth, migration, invasion and epithelial mesenchymal transition (EMT), as well as inhibition of cell apoptosis in many tumors including lung cancer [39–41]. Here, we reported that miR-134 is indeed suppressed in primary lung cancers compared with the matching adjacent normal tissues, and found 3′-UTR of the human CCND1 mRNA is really a target of miR-134. Collectively, we discovered that miR-134 inhibited NSCLC cell prolifferation, colony formation, migration and invasion, and promoted cell apoptosis by targeting 3′-UTR of *CCND1*.

## RESULTS

### MiR-134 is down-regulated in NSCLC tissues and cell lines, and benefits for prognosis

**Figure 1:**
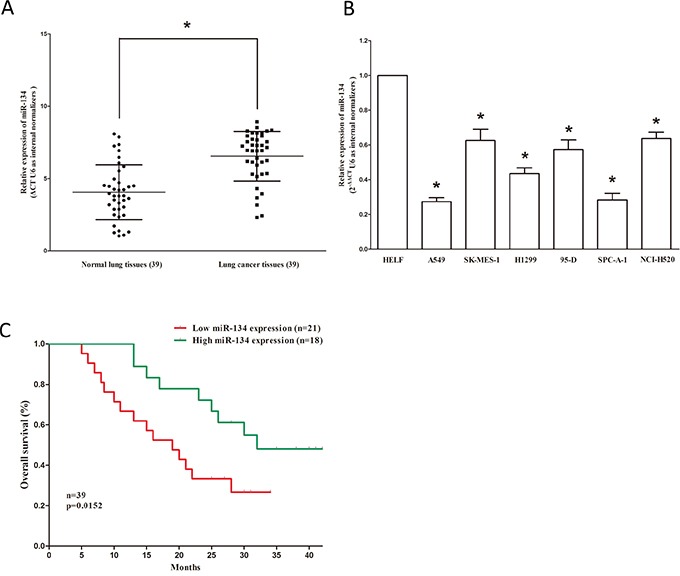
MiR-134 is down-regulated in primary human lung cancer and NSCLC cell lines, and benefits for prognosis **A.** miR-134 is significantly decreased in primary human lung cancer tissues in comparison to adjacent-normal lung cancer tissues. n=39 for each group. **B.** The expression level of miR-134 in six NSCLC cell lines and normal HELF cells. Assays were performed in triplicate. **C.** Kaplan-Meier survival analysis revealed that down-regulated miR-134 is associated with poor prognosis in patients with non-small cell lung cancer. *P < 0.001, Means ± SEM was shown. Statistical analysis was conducted using student t-test.

To determine whether miR-134 is down-regulated in lung cancer, we examined the miR-134 level in human primary lung tumors (NSCLC) and pair-matched adjacent lung normal tissues by qRT-PCR. We used U6 that is not deregulated in lung cancer for normalization. Results revealed that miR-134 expression in the tumors was significantly (*P*<0.05) reduced (mean=29% of decrease) in 39 lung cancers in comparison to their matched controls (Figure 1A). Next, we examined miR-134 expression in NSCLC cell lines, and results demonstrated a lower expression of miR-134 in A549, H1299, 95D, SK-MES-1, NCI-H520 and SPC-A-1 cell lines, in comparison to that of in human embryonic lung fibroblast (HELF), a normal lung cell line, (Figure 1B). In addition, to evaluate the clinical significance of miR-134, we assessed the correlation of its expression with clinic-pathological parameters (i.e., stage, maximum diameter and lymph node metastasis). Results demonstrated miR-134 expression levels in NSCLC were significantly associated with tumor size (*P* = 0.0003), smoking history (*P* = 0.0001), TNM stage (*P* = 0.0314), and lymph node metastasis (*P* = 0.0154). However, miR-134 expression was not correlated with other clinical characteristics such as differentiation (*P* = 0.1713), gender *(P* = 0.7062), age (*P* = 0.4877) or histological tumor type (*P* = 0.5273) in NSCLC (Table 1). Additionally, Kaplan–Meier survival analysis demonstrated that patients with low expression levels(≤29% of decrease, n=18) of miR-134 had shorter overall survival, in comparison to patients with high expression levels(>29% of decrease, n=21) of miR-134 (Figure 1C). These results demonstrated that down-regulation of miR-134 was associated with poor prognosis. Collectively, decreased expression of miR-134 might be a critical factor in NSCLC progression and development.

**Table 1:** Correlation between miR-134 expression and clinicopathological parameters of NSCLC patients (n=39)

Parameter	n	Relative NEAT1 expression		
		Low	High	*P*-value[^a^,^b^]
Age/years				0.4877
≤ 65	27	16	11	
> 65	12	5	7	
Gender				0.7062
Male	30	17	13	
Female	9	4	5	
Differentiation				0.1713
Well, moderate	11	8	3	
Poor	28	13	15	
Tumor size (maximum diametercm)				0.0003[*]
≤ 3cm	17	15	2	
> 3cm	22	6	16	
Smoking history				0.0001[*]
Smokers	21	5	16	
Never smokers	18	16	2	
Lymph node metastasis				0.0154[*]
Positive	26	18	8	
Negative	13	3	10	
TMN stage				0.0314[*]
I	11	9	2	
II/III/IV	29	12	17	
Histological tumor type				0.5273
Squamous cell carcinoma	19	9	10	
Adenocarcinoma	20	12	8	

a Chi-square test

b Fisher's exact test

* *P* < 0.05

### Expression of cyclin D1 is up-regulated in primary human lung cancer and negatively expressed related to miR-134

**Figure 2:**
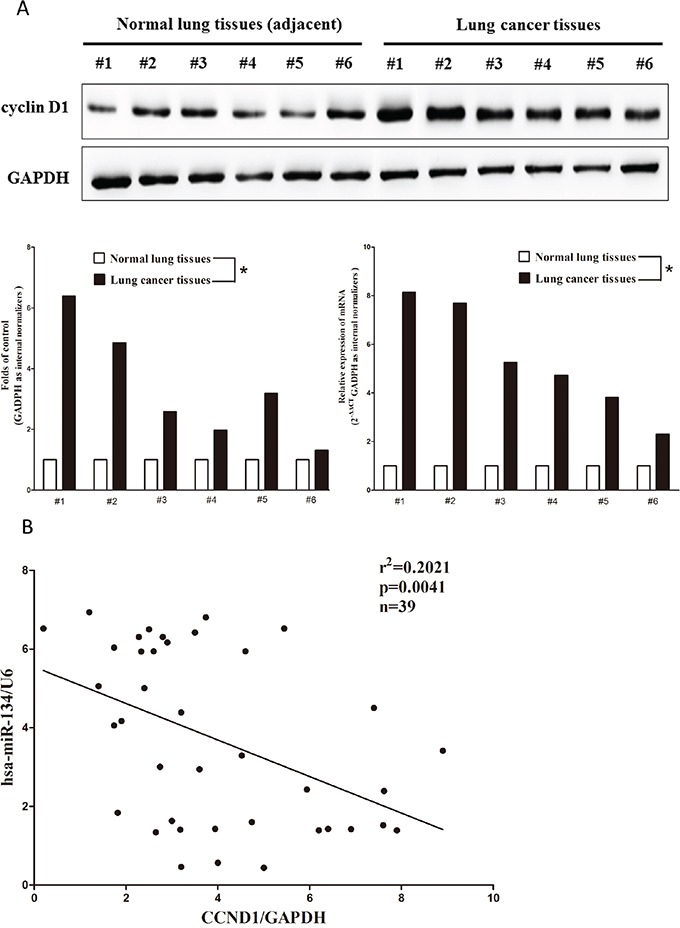
Expression of *CCND1* is up-regulated in primary human lung cancer and negatively expressed related to miR-134 **A.** Western-blot of cyclin D1 protein and qRT-PCR of CCND1 mRNA in lung cancer tissues and adjacent-normal lung cancers. n=39 for each group. **B.** Scatter plots showing the inverse association between miR-134 level and CCND1 mRNA expression. *P < 0.001, Means ± SEM was shown. Statistical analysis was conducted using student t-test.

cyclin D1 is important oncogene that shown strong power of oncogenicity, by promotion of cell growth, migration, invasion and epithelial mesenchymal transition (EMT), as well as inhibition of cell apoptosis in many tumors including lung cancer [39–41]. Thus, we next examined cyclin D1 expression in NSCLC and pair-matched adjacent lung tissues, and our western blot results demonstrated that cyclin D1 protein level was increased in lung cancer tissues in comparison to normal lung tissues (3.4-fold of increase) (Figure 2A). These results were confirmed by qRT-PCR of cyclin D1 mRNA expression (Figure 2A). Since cyclin D1 is the key role on regulation of cell cycle, aberrations of these three proteins might contribute to human lung cancer. Moreover, we assessed the correlation between CCND1 mRNA and miR-134 expression in 39 lung cancer tissues, and results indicated expression of CCND1 mRNA and miR-134 showed a remarkably inverse correlation as calculated by Pearson correlation (r^2^=0.2021, *P* =0.0041) (Figure 2B).

### MiR-134 targets human *CCND1*

**Figure 3:**
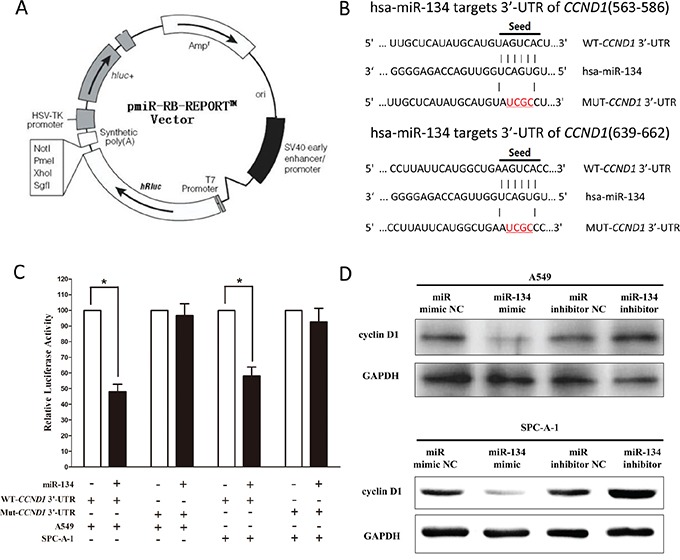
CCND1 proto-oncogene is a target of miR-134 at specific 3′-UTR sites **A.** pmiR-RB-REPORT TM dual-luciferase reporter vector. **B.** The 3′-UTR of *CCND1* harbors two miR-134 cognate sites. **C.** Relative luciferase activity of reporter plasmids carrying wild-type or mutant *CCND1* 3′-UTR in A549 and SPC-A-1 cells co-transfected with negative control (NC) or miR-134 mimic. **D.** Protein expression of cyclin D1 in A549 and SPC-A-1 cells after transfected with related miRNAs. Assays were performed in triplicate. *P < 0.001, Means ± SEM was shown. Statistical analysis was conducted using student t-test.

We then investigated the potential molecular mechanism of miR-134's antitumorigenic property in lung cancer cells. We searched different data bases (TargetScan, microRNA.org and PicTar) for its potential targets that exhibited oncogenic properties, and results demonstrated *CCND1* which harbored two conserved miR-134 cognate sites, namely, 563-586 and 639-662 of *CCND1* 3′-UTR was a predicted target of miR-134, (Figure 3B),. Next, we used luciferase reporter assays to determine whether *CCND1* expression are indeed regulated by miR-134, And results demonstrate that miR-134 inhibits luciferase activity by around 52% in A549 cells and 41% in SPC-A-1 cells when the reporter plasmid carried the WT *CCND1* 3′-UTR (Figure 3C), but no significant inhibition was observed at the reporter plasmid carried a mutant *CCND1* 3′-UTR. We next examined the role of miR-134 on the protein expression of cyclin D1. Our results of western blot demonstrated that miR-134 inhibited expression of cyclin D1 protein by approximately 80% and 85%, when compared with blank A549 and SPC-A-1 cells (Figure 3D), respectively. Our results reveal that miR-134 targets human *CCND1* by directly binding to the predicted sites in 3′-UTR of *CCND1* mRNA.

### Inhibition of miR-134 does not reverse the anticancer efficacy of silence of *CCND1* expression *in vitro*

**Figure 4:**
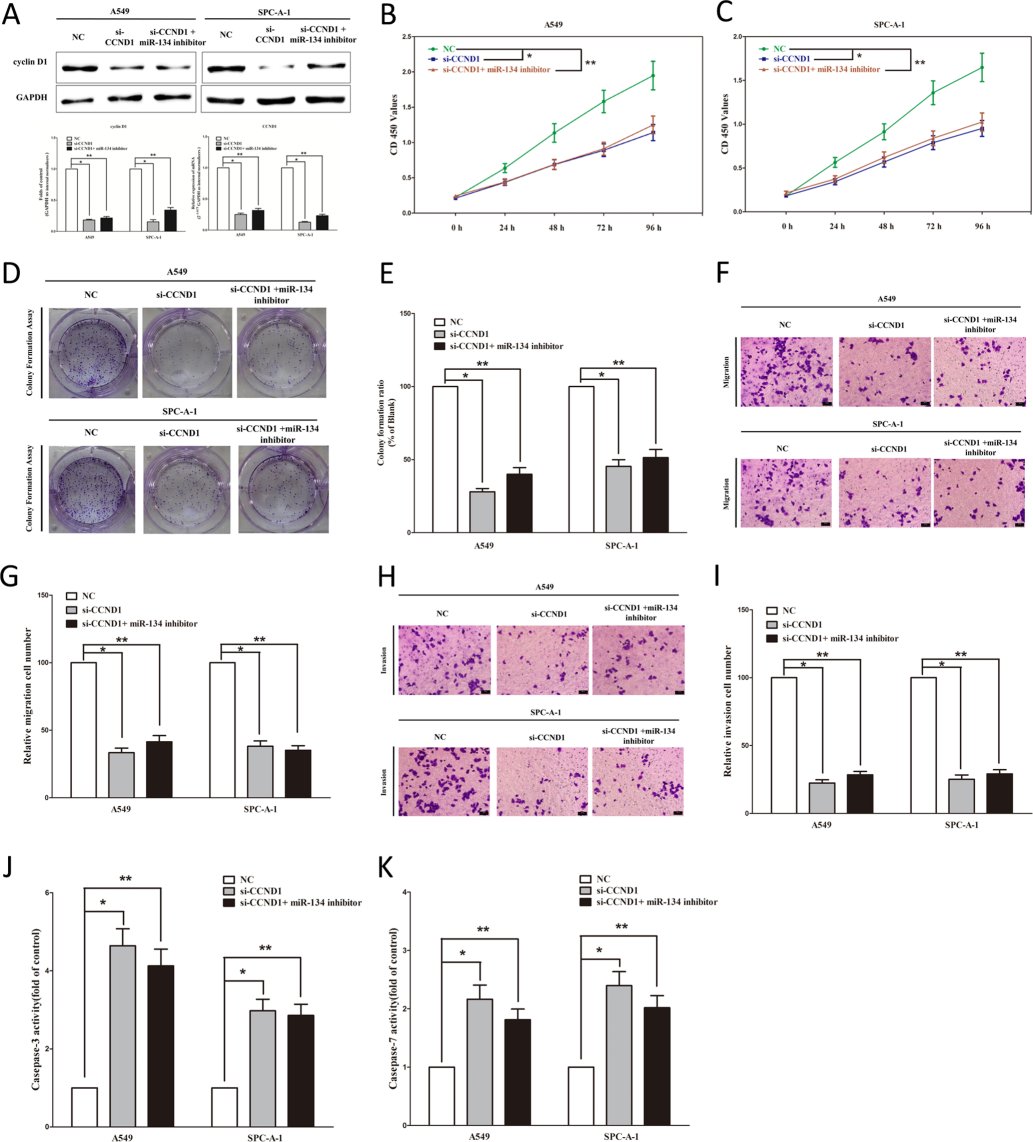
Inhibition of miR-134 does not reverse the anticancer efficacy of silence of CCND1 expression *in vitro* **A.** Western-blot of cyclin D1 protein and qRT-PCR of CCND1 mRNA in si-CCND1 treated and blank A549 and SPC-A-1 cells, and si-CCND1 + miR-134 inhibitor. **B-C.** CCK8 assays of A549 and SPC-A-1 cells after transfected (un-transfected) with si-CCND1 and si-CCND1 *+* miR-134 inhibitor. **D-E.** Shown are representative photomicrographs of colony formation assay after transfected with (without) si-CCND1 and si-CCND1 *+* miR-134 inhibitor for fourteen days. **F-G.** Shown are representative photomicrographs of transwell migration assay after transfected with (without) si-CCND1 and si-CCND1 *+* miR-134 inhibitor. **H-I.** Shown are representative photomicrographs of transwell invasion assay after transfected with (without) si-CCND1 and si-CCND1 *+* miR-134 inhibitor. **G-K.** Quantitative representation of caspase-3 and caspase-7 activity in A549 and SPC-A-1 cells transfected with (without) si-CCND1 and si-CCND1 *+* miR-134 inhibitor for forty eight hours. Assays were performed in triplicate. *P < 0.001, **P < 0.001, Means ± SEM was shown. Statistical analysis was conducted using ANOVA.

We next examined the potential tumorigenicity of *CCND1* in lung cancer. Silence of *CCND1* expression by si-CCND1 significantly inhibited the expression of *CCND1* (Figure 4A). Moreover, loss of *CCND1* expression also contributed to inhibition of NSCLC cell (both A549 and SPC-A-1 cells) growth (62% or 51% of decrease in A549 or SPC-A-1 cells) (Figure 4B–4E) and metastasis (58% or 55% of decrease in migration, 66% or 63% of decrease in invasion in A549 or SPC-A-1 cells) (Figure 4F–4I). In addition, inhibition of *CCND1* expression promoted apoptosis in lung cancer cell (4.5-fold of increase or 3.4-fold of increase in caspase 3 activity, 2.1-fold of increase or 2.3-fold of increase in caspase 7 activity inA549 or SPC-A-1 cells) (Figure 4J–4K). These results further verified the powerful tumorigenicity of *CCND1* in lung cancer. Thus, we adopted *CCND1* for as targeted oncogenes. However, inhibition of miR-134 does not reverse the anticancer efficacy of silence of *CCND1* expression in NSCLC cell lines (both A549 and SPC-A-1 cells). These results indicate that the anticancer efficacy of miR-134 is partly attributed to its inhibitory role on *CCND1*.

### MiR-134 suppresses tumor growth in nude mouse xenograft model

**Figure 5:**
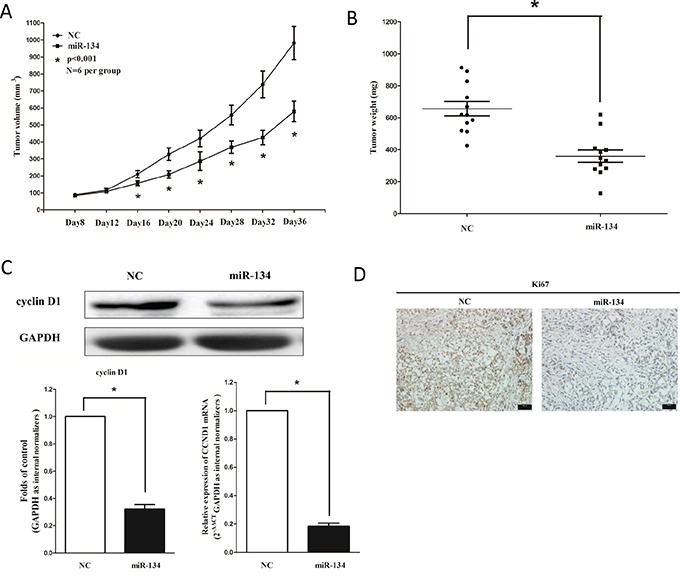
Ectopic expression of miR-134 suppresses tumor growth *in vivo* **A.** Tumor volume in nude mice. **B.** Tumor weight in nude mice. Each group contained six mice (n = 6); the data are presented as the mean ± SEM; *p < 0.001, compared with the NC group. **C.** The expression of cyclin D1 protein and mRNA in nude mice. Assays were performed in triplicate. *P < 0.001, Means ± SEM are shown. Statistical analysis was conducted using student t-test. **D.** Immunohistochemistry showed miR-134 decreased the proliferation index Ki67.

To validate the tumor suppressive efficiency of miR-134 *in vivo*, we established a BALB/c nude mouse xenograft model using A549 cells. The mice were treated as descript in method part. Our results demonstrated that the tumor volume and weight of mice treated with miR-134 mimic were significantly reduced (55% of decrease in tumor weight) relative to that of treated with miR mimic NC (Figures 5A and 5B). This result demonstrates miR-134 significantly suppresses the tumorigenicity of A549 cells in the nude mouse xenograft model. In addition, our results of western-blot and qRT-PCR demonstrated that the decreased expression (62% of decrease) of cyclin D1 in tumors developed from miR-134-mimic-treated nude mice relative to that of control tumors (Figure 5C). Moreover, immunohistochemical staining of resected tumor tissues found that tumors formed from miR-134-transfected A549 cells exhibited reduced positivity (72% of decrease) for Ki67 compared with those formed from control cells (Figure 5D). Thus, miR-134 reduces the growth of established NSCLC xenografts.

### MiR-134 inhibits lung cancer cell growth

**Figure 6:**
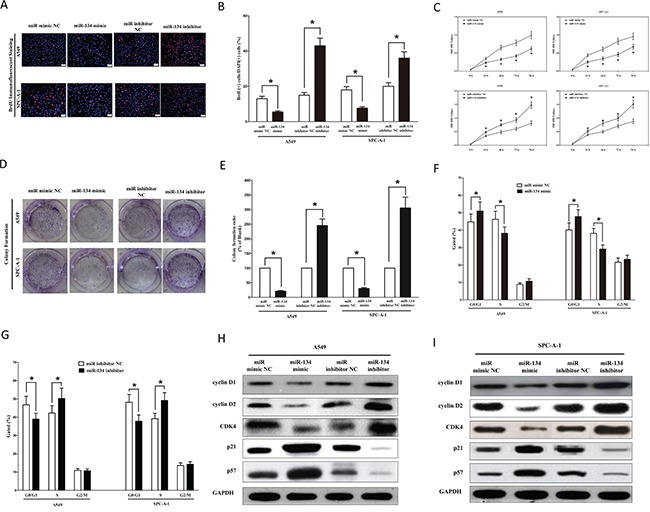
Ectopic expression of miR-134 inhibits proliferation and colony formation of A549 and SPC-A-1 cells **A.** Shown are representative photomicrographs of BrdU staining after transfected A549 and SPC-A-1 cells with miR-134 mimic, miR-134 mimic NC, miR-134 inhibitor or miR-134 inhibitor NC for twenty four hours. Bar = 100 μm. **B.** Statistical analysis of BrdU staining. **C.** CCK8 assays of A549 and SPC-A-1 cells after transfected with miR-134 mimic, miR-134 mimic NC, miR-134 inhibitor, miR-134 inhibitor NC. **D.** Shown are representative photomicrographs of colony formation assay after transfected with miR-134 mimic, miR-134 mimic NC, miR-134 inhibitor or miR-134 inhibitor NC for fourteen days. **E.** Statistical analysis of colony formation assay. Assays were performed in triplicate. **F-G.** Cell-cycle analysis was performed forty eight hours following the treatment A549 and SPC-A-1 cells with miR-134 mimic or miR-134 mimic NC, miR-134 inhibitor or miR-134 inhibitor NC. The DNA content was quantified by flow cytometric analysis. **H-I.** Expression of cyclin D1, cyclin D1, CDK4, p21 and p57 protein in transfected A549 and SPC-A-1 cells. Assays were performed in triplicate. *P < 0.001, Means ± SEM was shown. Statistical analysis was conducted using student t-test.

To further explore its anticancer efficacy on miR-134 in NSCLC, we examined the role of miR-134 on NSCLC cell (A549 and SPC-A-1) proliferation. Our results of BrdU staining revealed that miR-134 inhibited A549 and SPC-A-1 cell DNA synthesisbyapproximately48% (Figures 6A and 6B) and 52% (Figures 6A and 6B), compared with blank A549 and SPC-A-1 cells, respectively. However, miR-134 inhibitor treatment increased A549 and SPC-A-1 cell DNA synthesis by approximately 2.2 folds (Figures 6A and 6B) and 1.5 folds (Figures 6A and 6B) compared with blank A549 and SPC-A-1 cells, separately. To verify these results, we also did the CCK8 assay, and results demonstrated that miR-134 over-expression significantly attenuated A549 and SPC-A-1 cells vitality, while loss of miR-134 promoted cell proliferation (Figure 6C). In addition, we used colony formation assay to investigate the role of miR-134 on clonogenic survival, and results demonstrated miR-134 mimic treatment caused a decrease in the clonogenic survival of A549 and SPC-A-1 cells compared with blank A549 and blank SPC-A-1 cells (Figures 6D and 6E), while miR-134 inhibitor-treated A549 cells showed an significant increase in the clonogenic survival, when compared with blank A549 and blank SPC-A-1 cells (Figures 6D and 6E). Furthermore, the growth inhibitory role of miR-134 on A549 and SPC-A-1 cell lines was accompanied by a corresponding decrease in the proportion of cells in the S phase and an increase in the proportion of cells in G1 (Figure 6F–6G).

We next examined the efficiency of miR-134 on expression of cyclin D1. Our results discovered that miR-134 significantly inhibited the protein expression of cyclin D1, while loss of miR-134 remarkably increased the level of cyclin D1 in A549 and SPC-A-1 cells (Figures 6H and 6I). Cyclin D2 is highly expressed and promotes tumorigenesis in numerous tumors [44, 45]. In our research, the protein expression of cyclin D2 was repressed by over-expression of miR-134 (Figures 6G and 6H). Over-expression of CDK4 has been discovered in numerous of malignant neoplasms, including glioma, breast cancer, and lung cancer [46]. In our research, the protein expression of CDK4 was repressed by over-expression of miR-134 in A549 and SPC-A-1 cells (Figures 6G and 6H). Our study revealed that the over-expression of miR-134 is a mechanism for the up-regulation of p57 level in NSCLC cell lines (A549 and SPC-A-1) (Figures 6G and 6H). Transfection of p21 (a cell cycle inhibitor) expressive constructs into normal [50] and tumor cell lines [51] leads to cell cycle arrest in G1 [52]. Our study revealed that miR-134 up-regulated p21 level in NSCLC cell lines (A549 and SPC-A-1) (Figures 6G and 6H).

Collectively, these results clearly demonstrated that miR-134 markedly repressed cell growth in lung cancer cells.

### MiR-134 inhibits lung cancer cell metastasis

Then, we explored the role of miR-134 on A549 and SPC-A-1 cells migration and invasion. Invasion and migration through the basement membrane are characteristics of metastatic cancer cells.

**Figure 7:**
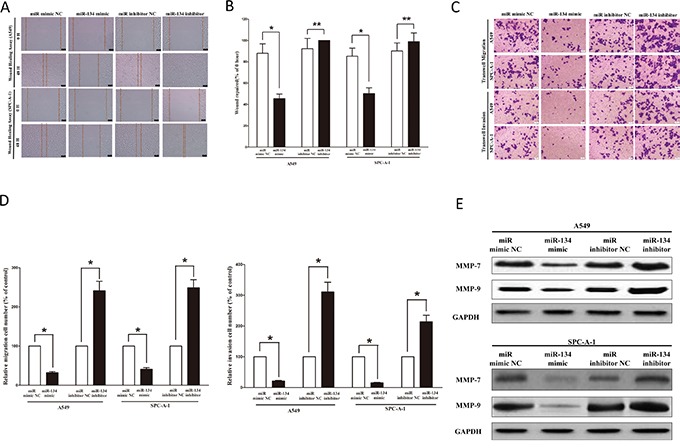
Ectopic expression of miR-134 in A549 and SPC-A-1 cells reduces cell migration and invasion motility **A.** Shown are representative photomicrographs of “wound healing assay” in A549 and SPC-A-1 cells after transfected miRNAs for 0 hour and forty eight hours. Bar = 100 μm. **B.** Statistical analysis of “wound healing assay”. **C.** A549 and SPC-A-1 cells were loaded onto the top well of a transwell inserts for cell migration or invasion assay. After twenty four hours, cells that migrated to the bottom chamber containing serum-supplemented medium were stained with 0.1% crystal violet, visualized under a phase-contrast microscope, and photographed. Bar = 100 μm. **D.** Total number of cells in five fields was counted manually. **E.** Expression of MMP-7 and MMP-9 protein in A549 and SPC-A-1 cells after transfection. Assays were performed in triplicate. *P < 0.001, Means ± SEM was shown. Statistical analysis was conducted using student t-test.

We used two different approaches to assess the role of miR-134 on the ability of A549 and SPC-A-1 cells migration. In the first technique, we used a “scratch wound healing” assay. Motility of cells at different time points after generation of the wound was monitored under a microscope. Closure of the wound was complete within forty eight hours in control A549 and SPC-A-1 cells (Figures 7A and 7B). In contrast, miR-134-expressing cells migrated toward the wound at a much slower (58% of distance or 50% of distance in A549 or SPC-A-1 cells) rate (Figures 7A and 7B). In the second approach, we used transwell/migration assay to assess the role of miR-134 on cell migration. As expected, migration of miR-134-expressing clones was inhibited by 63% in A549 and 54% in SPC-A-1 cells, in comparison to the blank A549 and SPC-A-1 cells (Figures 7C and 7D), respectively. However, when treated with miR-134 inhibitor, migration in miR-134-expression defect A549 and SPC-A-1 cells were significantly increased by approximately 2.3 and 2.5 folds relative to blank A549 and SPC-A-1 cells (Figures 7C and 7D) respectively.

To investigate the role of miR-134 on A549 and SPC-A-1 cells invasion, we used a transwell invasion assay. As expected, invasion of miR-134-expressing clones was inhibited by 66% in A549 and 73% in SPC-A-1 cells, relative to the blank A549 and SPC-A-1 cells (Figures 7C and 7D), respectively. However, when treated with miR-134 inhibitor, invasion in miR-134-expression defect A549 and SPC-A-1 cells were significantly increased by approximately 3.2 and 2.1 folds relative to blank A549 and SPC-A-1 cells (Figures 7C and 7D), separately.

We also investigated the role of miR-134 on expression of MMP-7 and MMP-9, which all play a key role on tumor metastasis, and results indicated miR-134 inhibited the protein expression of MMP-7 and MMP-9 both in A549 and SPC-A-1 cells (Figure 7E). As expected, loss of miR-134 significantly increased the protein expression of MMP-7 and MMP-9 in both A549 and SPC-A-1 cells (Figure 7E).

These results, taken together, clearly demonstrated that miR-134 expression markedly reduces the migration and invasion motility of lung cancer cells.

### MiR-134 promotes lung cancer cell apoptosis

**Figure 8:**
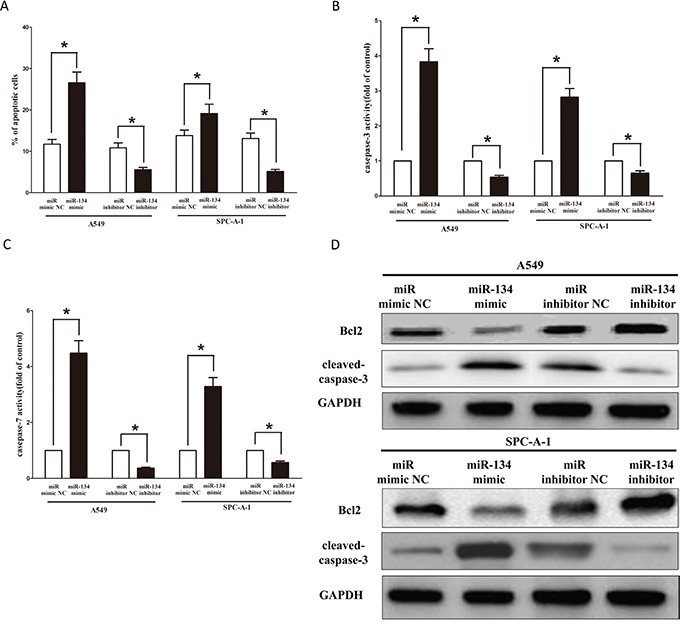
Ectopic expression of miR-134 promotes apoptosis in A549 and SPC-A-1 cells **A.** Shown are statistical analysis of flow cytometric analysis. **B-C.** Quantitative representation of caspase-3 and caspase-7 activity in A549 and SPC-A-1 cells transfected with related miRNAs for forty eight hours. **D.** Western-blot of Bcl2 protein in A549 and SPC-A-1 cells after transfection. Assays were performed in triplicate. *P < 0.001, Means ± SEM was shown. Statistical analysis was conducted using student t-test.

Next, we examined the role of miR-134 on A549 and SPC-A-1 cells apoptosis. Our results of flow cytometric analysis demonstrated that forced expression of miR-134resulted in a ~2.4 folds and ~1.5 folds of increase in apoptotic cell death of A549 and SPC-A-1 cells (Figure 8A), respectively. However, the percentage of apoptotic cells induced by miR-134 was decreased to the basal level when the cells were treated with the specific miR-134 inhibitor (Figure 8A). In addition, we also tested the caspase-3 and caspase-7 activity after treatment of A549 and SPC-A-1 cells with miR-134 mimic or miR-134 mimic NC, miR-134 inhibitor or miR-134 inhibitor NC, and results showed that miR-134 significantly increased the caspase-3 and caspase-7 activity in A549 and SPC-A-1 cell lysate, by approximately 3.7 and 4.3 folds increase (caspase-3 activity), 2.9 and 3.6 folds increase (caspase-7 activity), than that of in bank A549 and SPC-A-1 cells (Figures 8B and 8C), respectively. However, loss of miR-134 by transfecting with miR-134 inhibitor remarkably reduced the caspase-3 and caspase-7 activity in A549 and SPC-A-1 cell lysate, compared with that of in bank A549 and blank SPC-A-1 cells (Figures 8B and 8C), respectively. Moreover, miR-134 also inhibited the expression level of anti-apoptotic protein Bcl2 (Figure 8D), and increased the protein expression of cleaved-caspase-3 (Figure 8D) in A549 and SPC-A-1 cells. These results demonstrated that miR-134 indeed promoted apoptosis in A549 and SPC-A-1 cells.

## DISCUSSION

Our present study has revealed the following novel findings: (i) exogenously overexpressed miR-134 suppresses tumor regeneration in 6 lung cancer xenograft models and inhibits cell growth *in vitro* and *in vivo*; (ii) miR-134 overexpression inhibits NSCLC cells migration and invasion; (iii) inhibition of miR-134 in NSCLC cells results in high clonal, clonogenic, and tumorigenic properties; (iv) miR-134 overexpression promotes NSCLC cells apoptosis, and inhibition of miR-134 inhibits NSCLC cell apoptosis; (v) miR-134 targets *CCND1* in NSCLC cells and negatively expressed with *CCND1*.

Up to date, the molecular mechanisms about NSCLC progression sparsely elaborated. Hence, a better understanding of the molecular mechanisms referred to tumor formation and development will be helpful to exploit novel therapeutic strategies and targets for treatment of human lung cancers. Although dysregulation of miRNAs was reported in numerous of human cancers [53], aberrant expression and potential role of miRNAs in lung cancers were under studied. Decreased expression of miR-134 has been reported by miRNA profile studies on melanoma [25], breast cancer [26, 27], ovarian cancer [28], renal cell carcinoma [29], endometrial cancer [30], embryonal carcinoma [31], hepatocellular carcinoma [32, 33], head and neck carcinoma [34] and glioblastoma [35]. Our results also indicated that miR-134 was decreased in NSCLC tissues and cells, indicating disorder of miR-134 was an early event of NSCLC tumorigenesis. We therefore explored the speculative tumor suppressive efficiency of miR-134 in human lung cancer cell lines. Firstly, we examined the mechanism of miR-134 on lung cancer cell growth, and found that restoration of miR-134 in the lung cancer cell lines A549 and SPC-A-1 significantly suppressed cell growth as evidenced by colony formation assays, BrdU, and cell viability (CCK8). The growth-inhibitory role of miR-134 may be attributed to the fact thatmiR-134 targets 3′-UTR of CCND1 mRNA, and inhibits the expression of CCND1 in lung cancer cells. In addition, miR-134 also inhibited cyclin D1, cyclin D2 and promoted p57 and p21 expression levels in lung cancer cells, which further contributed to the growth-delay efficacy of miR-134. In addition to inhibition of cell growth, its growth-inhibitory role was also associated with its promotion on cell apoptosis. We discovered that miR-134 induced cell apoptosis happens by the regulation of extrinsic apoptosis pathway, which had been viewed as a crucial antitumor mechanism [54–56]. The expression of critical anti-apoptosis protein Bcl2 was decreased after transfecting with miR-134, and the activity of its downstream factor, active apoptosis executor caspase-3 was up-regulated, leading to initiate a caspase cascade, and finally resulted in cell apoptosis [57].

Restoration of miR-134 suppressed the cell metastasis *in vitro* assays. The reduced disseminating efficiency and cell motility induced by miR-134 in NSCLC cell lines were demonstrated to be related to decreased protein expression of cell migration and invasion molecules matrix metalloproteinases 7 (MMP-7) and matrix metalloproteinases (MMP-9). MMP-7 and MMP-9 are members of the matrix metalloproteinases (MMPs) family, which are located in extracellular and control basic cellular processes, such as morpho-genesis, migration and survival, and they can degrade extracellular matrix during the cancer metastatic process [58, 59]. MMP-7 is an assured rabble-rouser of aggressive behavior in numerous of cancers including CRC. MMP-9 is established as a crucial module for initiating of the pre-metastatic niche [57]. Thus, decreased expression of MMP-7 and MMP-9 by miR-134 led to alleviated cell metastasis ability.

Having shown the critical effects of miR-134 on suppressing NSCLC progression, we explored the potential gene effectors involving in its function. Amazingly, a single miRNA could affect numerous of target genes coincidently [43], for instance, it has been reported that miR-134 suppresses progression of melanoma by down-regulating CD86 [25]; and miR-134 could modulate resistance to doxorubicin in human breast cancer cells by repressing ABCC1 oncogene [27]. Importantly, Chen *et al* revealed miR-134 regulated SCLC cell growth and apoptosis by targeting WWOX gene and suppressing the ERK1/2 signaling pathway [36], and Li *et al* discovered miR-134 inhibits epithelial to mesenchymal transition by targeting FOXM1 in non-small cell lung cancer cells [38], but among the miRNAs predicted to target genes, we found that cyclin D1 acts as a critical effector of miR-134. But among the predicted target genes of miR-134, we discovered CCND1 acted as a crucial effector of miR-134. Our results demonstrated miR-134 could significantly inhibit the luciferase activity of wide type of Luc-*CCND1*-3′-UTR by directly binding with the targeted sites of the 3′-UTR in CCND1 mRNA. Therefore we selected CCND1 as the target gene, and focused on it for further analysis.

In our present study, we discovered miR-134 was an underlying prognostic factor for NSCLC, and found miR-134 is remarkably decreased in human NSCLC tissues in comparison to normal lung tissues. Moreover, we also revealed over-expression of miR-134 inhibits cell growth, metastasis, and promotes cell apoptosis in NSCLC cell lines, through directly targeting CCND1. The present results elucidate a potential mechanism underlying the tumor-suppressor role of miR-134, and indicate that miR-134 could be a useful marker and potential therapeutic target in NSCLC.

## MATERIALS AND METHODS

### Tissue collection

Lung cancer tissues and adjacent normal lung tissues were obtained from 39 patients who had undergone surgery at the People's Hospital of Wuhan University, between 2006 and 2010 and who were diagnosed with lung cancer based on histopathological evaluation. No local or systemic treatment had been conducted in these patients before the operation. All the tissue samples were collected, immediately snap frozen in liquid nitrogen, and stored at −80°C until RNA extraction. The study was approved by the Research Ethics Committee of Wuhan University (Wuhan, Hubei, PR China). Informed consent was obtained from all patients.

### Cell culture and transfection

The human NSCLC cell lines, namely, A549, SPC-A-1, H1299, SK-MES-1, NCI-H520 and 95D cells were grown in RPMI 1640 (Gibco, USA), and HELF cells were grown in DMEM medium containing 10% heat-inactivated (56°C, 30 min) fetal calf serum, 2 mmol/L glutamine, penicillin (100 U/mL) and streptomycin (100 U/mL), which was maintained in an incubator at 37°C with 5% CO_2_ in a humidified atmosphere. Hsa-miR-134 mimic and mimic negative control, hsa-miR-134 inhibitor and inhibitor negative control were purchased from GenePharma Co., Ltd. (Shanghai, China). For convenience, hsa-miR-134 mimic and mimic negative control, hsa-miR-134 inhibitor and inhibitor negative control were simply referred to as miR-134 mimic and miR mimic NC, miR-134 inhibitor and miR inhibitor NC, respectively. Complete medium without antibiotics was used to culture the cells at least twenty-four hours prior to transfection. The cells were washed with 1× PBS (pH7.4) and then transiently transfected with 50 nM miR-134 mimic or miR mimic NC, 100 nM miR-134 inhibitor or miR inhibitor NC, using Lipofectamine™ 2000 (Invitrogen, Carlsbad, CA, USA) according to the manufacturer's instructions.

### Western blot analysis

Forty-eight hours after transfection, total protein was extracted from the A549 and SPC-A-1 cells using RIPA cell lysis reagent containing proteinase and phosphatase inhibitors (Sangon Biotech, Shanghai, China) at 4°C for 30 min. Cell lysates were centrifuged at 12,000× g for 20 min at 4°C, and the protein concentrations of the supernatant were determined using the BCA protein assay reagent kit (Thermo). The supernatants containing total protein were then mixed with a corresponding volume of 5× SDS loading buffer and heated at 100°C for 10 min. Then, the supernatant lysates were run on 10% SDS-polyacrylamide gels (50 μg/lane), and proteins were transferred to poly (vinylidene fluoride) (PVDF) membranes (Hertfordshire, UK) by semidry electroblotting (1.5 mA/cm2). PVDF membranes were then incubated in blocking buffer [Tris-buffered saline (TBS) supplemented with 0.05% (vol/vol) Tween 20; TBST] containing 5% (wt/vol) skimmed milk powder for 120 min at room temperature followed by three 10 min washes in TBST. The PVDF membranes were then incubated with anti-cyclin D1 (1:1000 dilutions, Affinity), anti-cyclin D2 (1:1000 dilutions, Affinity), anti-CDK4 (1:1000 dilutions, Affinity), anti-p21 (1:1000 dilutions, Affinity), anti-p57 (1:1000 dilutions, Affinity), anti-MMP-7 (1:1000 dilutions, Affinity), anti-MMP-9 (1:1,000 dilutions, Affinity), anti-cleaved caspase 3 (1:1,000 dilutions, Affinity) and anti-GADPH (1:5,000 dilutions, Affinity) as internal normalizers in TBST containing 5% (wt/vol) skimmed milk powder (antibody buffer) overnight at 4°C on a three-dimensional rocking table. Then the membranes were washed three times for 10 min in TBST and then incubated with goat anti-rabbit IgG conjugated to horseradish peroxidase (1:12,000 dilutions) in antibody buffer for 120 min. Finally, membranes were washed three times for 10 min in TBST and exposed to ECL Advance reagent (GE Healthcare Biosciences, Buckinghamshire, UK) for 2 min as described in the manufacturer's protocol. Then membranes were exposed to Hyperfilm-ECL (GE Healthcare Bio-Sciences) for 2–5 min and visualized using a Fluor S Multimager and Quantity One 4.1 (Bio-Rad Laboratories, Hercules, CA). The molecular weights of the bands were calculated by a comparison with prestained molecular weight markers (molecular weight range: 6,500–250,000) that were run in parallel with the samples. Semiquantitative analysis of specific immunolabeled bands was performed using a Fluor S image analyzer and Quantity One 4.1.

### RNA isolation and quantitative reverse transcription polymerase chain reaction (qRT-PCR)

Total RNA from the cultured cells was extracted using Trizol reagent (Invitrogen) according to the manufacturer's instructions. MiRNA levels were measured by qRT-PCR. For the qRT-PCR detection of mature miR-134 expression, we purchased the Bulge-Loop™ miRNA qRT-PCR Primer Set and the miRNA qRT-PCR Control Primer Set (both from RiboBio). RNA (2 μg) was converted into cDNA using the PrimeScript™ RT reagent kit with gDNA Eraser (Takara, Dalian, China) according to the manufacturer's instructions. qRT-PCR was performed using SYBR^®^ Premix Ex Taq™ II (Takara) in the ABI PRISM® 7300 real-time PCR system (Applied Biosystems, Foster City, CA, USA). GADPH and U6 were used as endogenous controls. In addition, melting curves were used to evaluate non-specific amplification. The relative expression level was calculated using the 2^−ΔΔCt^ method. The primer sequences used in this study are as follows: the primers of miR-134 were purchased from RiboBio (RiboBio Co., Ltd, Guangzhou, China); Primer sequences for miR-134 and U6 are shown in Table S1. Human CCND1: sense: 5′-CTCCTCTCCGGAGCATTTTGATA-3′, antisense: 5′-TTAAAGACAGTTTTTGGGTAATCT-3′; human MMP-7: sense: 5′-GAGTGCCAGATGTTGCAGAA-3′, antisense: 5′-AAATGCAGGGGGATCTCTTT-3′; human MMP-9: sense: 5′-CTGCAGTGCCCTGAGGACTA-3′, antisense: 5′-ACTCCTCCCTTTCCTCCAGA-3′; The formula and its derivations were obtained from the ABI Prism 7300 sequence detection system user guide. Statistical analysis was performed on the fold change.

### Colony formation assay

Cells were transfected with miR-134 mimic or miR mimic NC, miR-134 inhibitor or miR inhibitor NC, as described above. Twenty-four hours later, transfected cells were trypsinized, counted and replated at a density of 500 cells/6 cm dish. Ten days later, colonies resulting from the surviving cells were fixed with 3.7% methanol, stained with 0.1% crystal violet and counted. Colonies containing at least 50 cells were scored. Each assay was performed in triplicates.

### Luciferase reporter assays

The 3′-untranslated region (UTR) of human *CCND1* was amplified from human genomic DNA and individually inserted into the pmiR-RB-REPORT™ (Ribobio, Guangzhou, China) using the XhoI and NotI sites. Similarly, the fragment of *CCND1* 3′-UTR mutant was inserted into the pmiR-RB-REPORT™ control vector at the same sites. For reporter assays, A549 cells were co-transfected with wild-type (mutant) reporter plasmid and miR-134 mimics (miR mimic NC) using Lipofectamine 2000 (Invitrogen). Firefly and Renilla luciferase activities were measured in cell lysates using the Dual-Luciferase Reporter Assay system. Luciferase activity was measured forty-eight hours post-transfection using dual-glo luciferase reporter system according to the manufacturer's instructions (Promega, Madison, WI, USA). Firefly luciferase units were normalized against Renilla luciferase units to control for transfection efficiency.

### Transwell migration/invasion assay

A549 and SPC-A-1 cells were grown in RPMI 1640 containing 10% fetal bovine serum to ~60% confluence and transfected with 50 nM miR-134 mimic or a negative control, 100 nM miR-134 inhibitor or a negative control. After twenty-four hours, the cells were harvested by trypsinization and washed once with Hanks' balanced salt solution (Invitrogen). To measure cell migration, 8-mm pore size culture inserts (Transwell; Costar, High Wycombe, UK) were placed into the wells of 24-well culture plates, separating the upper and the lower chambers. In the lower chamber, 500 μL of RPMI 1640 containing 10% FBS was added. Then, serum-free medium containing 5× 10 ^4^ cells were added to the upper chamber for migration assays, whereas 1×10^5^ cells were used for matrigel invasion assays. After twenty-four hours of incubation at 37°C with 5% CO_2_, the number of cells that had migrated through the pores was quantified by counting 10 independent visual fields under the microscope (Olympus) using a ×20 magnifications, and cell morphology was observed by staining with 0.1% crystal violet. Filters were washed thoroughly with 1× PBS and dissolved in 500 μL of 33% acetic acid, and absorbance was measured at 570 nm. Absorbance of cells incubated in the serum-free medium in the bottom chamber was used as negative control. Each experiment was performed at least three times.

### BrdU immunofluorescence assay

A549 and SPC-A-1 cells were seeded on sterile cover glasses placed in the 6-well plates. After transfection with miR-134 mimic, miR mimic NC, miR-134 inhibitor, miR inhibitor NC for forty eight hours, the BrdU (5-bromo-2-deoxyuridine; Sigma) stock solution at 10 mg/mL in saline was diluted 1000× in the culture medium and incubated for 60 min. After washing with 1× PBS, cells were then fixed for 20 min in 4% paraformaldehyde (PFA) and permeabilized with 0.3% Triton X-100 for 10 min. After blocking with 10% goat serum in 1× PBS for 1 h, cells were incubated with a primary rabbit antibody against BrdU (1:200, Abcam) over night at 4°C, and then incubated with the secondary antibody coupled to a fluorescent marker, Cy3, at room temperature for 2 h. After DAPI staining and 1× PBS washing, the cover slips were mounted on to glass slides with anti-fade solution and visualized using a fluorescence microscope (Olympus 600 auto-biochemical analyzer, Tokyo, Japan) with Image-Pro Plus software for image analysis, and 10 microscopic fields were taken for calculating BrdU.

### CCK8 assay

Cell growth was measured using the cell proliferation reagent WST-8 (Roche Biochemicals, Mannheim, Germany). After plating cells in 96-well microtiter plates (Corning Costar, Corning, NY) at 1.0× 10^3^ /well, 10 μL of CCK8 was added to each well at the time of harvest, according to the manufacturer's instructions. One hour after adding CCK8, cellular viability was determined by measuring the absorbance of the converted dye at 450 nm.

### Transfection of siRNA

cyclin D1 siRNA was purchased from Santa Cruz (sc-29286). For transfection, the cells were plated on an antibiotic-free growth medium at 30–40% confluence approximately 24 h before transfection. RNA oligonucleotides were transfected using Lipofectamine™ 2000 (Invitrogen, USA) according to the manufacturer's protocol.

### Tumor formation in BALB/c nude mice

BALB/c athymic nude mice (male, 4–6-weeks old and 16–20 g) were purchased from Hubei Research Center of Laboratory Animal (Wuhan, China). All animal experiments were carried out in accordance with the Guide for the Care and Use of Laboratory Animals of Wuhan University. To establish lung cancer xenograft model, 5× 10^5^ A549 cells were suspended in 100 μL phosphate-buffered saline and inoculated subcutaneously into the flanks of nude mice. After 8 days, the transplanted nude mice were randomly divided into two groups (n=6 each). miR-134 mimic (miR-134) or miR mimic NC (NC) (RiboBio Co., Ltd, Guangzhou, China) was directly injected into the implanted tumor at the dose of 1 nmol (in 20 μL phosphate-buffered saline) per mouse every 4 days for seven times. The tumor size was monitored by measuring the length (L) and width (W) with calipers every 4 day, and the volumes were calculated using the formula: (L × W^2^)/2. Mice were killed by cervical dislocation in day 28, and the tumors were excised and snap-frozen for protein and RNA extraction.

### Immunohistochemistry

Immunohistochemistry of the tumor tissues was performed as described previously [60–64]. 3-μm tumor sections were incubated with commercial rabbit polyclonal antibodies against Ki67 (Affinity) at 1/100 dilution overnight at 4°C. Then, the sections were conjugated with horseradish peroxidase (HRP) antibody (1:500 dilution; Santa Cruz Biotechnology, Santa Cruz, CA) at room temperature for 2 h, then covered by DAB (Vector Laboratories, Burlingame, CA), and slides were mounted with Vectashield mounting medium (Vector Laboratories). Subsequently, all fields were observed under light microscopy (Olympus 600 auto-biochemical analyzer, Tokyo, Japan). Control experiments without primary antibody demonstrated that the signals observed were specific.

### Flow cytometry

#### Apoptosis analysis

A549 and SPC-A-1 cells transfected with miR-134 mimic or negative control were trypsinized and resuspended in 1× binding buffer at 1× 10^6^ cells/mL. 100 μL of this cell suspension was incubated with 5 μL of FITC-Annexin V and 5 μL propridium iodide (PI) for 15 minutes in the dark. The reaction was terminated with the addition of 400 μL 1× binding buffer and analyzed with FACSCalibur using the CellQuest software (Becton Dickinson). FITC-Annexin V-positive and PI-negative cells were considered as apoptotic and the experiments were carried out in triplicates.

#### Cell-cycle analysis

Transfected cells were harvested forty-eight hours after transfection. The cells were fixed in 70% ethanol, washed once with PBS, and then labeled with propidium iodide (Sigma-Aldrich) in the presence of RNase A (Sigma-Aldrich) for 30 min in the dark (50 g/mL). Samples were run on a FACSalibur flow cytometer (Becton-Dickinson, FL, NJ, USA), and the percentages of cells within each phase of the cell cycle were analyzed using Cell Quest software.

### Wound healing assay *in vitro*

The A549 and SPC-A-1 cells were seeded in 6-well plates and incubated for twenty-four hours. Then a linear wound was tehncreated by dragging a 100-μL pipette tip through the monolayer prior to transfection. Cellular debris was removed by gentle washes with culture medium, following which transfection was performed immediately, and the cells were allowed to migrate for a further forty-eight hours. The healing process was dynamically photographed after the wound was introduced using a microscope (Olympus 600 auto-biochemical analyzer, Tokyo, Japan). Migration distance was measured from images (5 fields) taken at each indicated time point. The gap size was analyzed using Image-Pro Plus 6.0 software. The residual gap between the migrating cells from the opposing wound edge was expressed as a percentage of the initial gap size.

### Caspase-3/7 activity assay

The activity of caspase-3/7 was determined using the caspase-3/7 activity kit (Beyotime Institute of Biotechnology, Haimen, China). To evaluate the activity of caspase-3/7, cell lysates were prepared after their respective treatment with various designated treatments. Assays were performed on 96-well microtitre plates by incubating 10 μL protein of cell lysate per sample in 80 μL reaction buffer (1% NP-40, 20 mM Tris-HCl (pH 7.5), 137 mM Nad and 10% glycerol) containing 10 μL caspase-3 substrate (Ac-DEVD-pNA) (2 mM). Lysates were incubated at 37°C for 4 h. Samples were measured with an ELISA reader at an absorbance of 405nm. The detail analysis procedure was described in the manufacturer's protocol.

### Statistical analysis

All experiments were repeated 3 times independently. The results are presented as the means ± standard error mean (SEM). Two independent sample t-test or One-Way Analysis of Variance (ANOVA) was performed using SPSS 19.0 software in order to detect significant differences in measured variables among groups. A value of *P* <0.05 was considered to indicate a statistically significant difference.

## SUPPLEMENTARY TABLES



## Nonstandard abbreviations

miR: microRNA
miR-134: hsa-microRNA-134
NSCLC: non-small cell lung cancer
SCLC: small cell lung cancer
3′-UTR: 3′-untranslated region
nt: nucleotide
OS: overall survival
