# Supplementary material for: Hsa-miR-134 suppresses non-small cell lung cancer (NSCLC) development through down-regulation of CCND1
Source: Oncotarget. 2016 Jun 14;7(24):35960–78. doi: 10.18632/oncotarget.8482 (PMC5094975; doi:10.18632/oncotarget.8482)
Supplement: Supplementary file 1 [file oncotarget-07-35960-s001.pdf]

Hsa-miR-134 suppresses non-small cell lung cancer (NSCLC) development through down-regulation of CCND1

SUPPLEMENTARY TABLE

Supplementary Table S1: Primer sequences for quantitative reverse transcription(RT)-PCR

Name	Accession	Sequence
hsa-miR-134-5p	MIMAT0000447	RT-premier:5′-GTCGTATCCAGTGCAGGGTCCGAGGTATTCGCACTGGATACGACCCCCTC-3′
		Sense:5′- ACGGGCTGTGACTGGTTGACTA-3′
		Antisense:5′-CGCAGGGTCCGAGGTATTC-3′
U6	NR_004394	RT-premier:5′-AACGCTTCACGAATTTGCGT-3′
		Sense:5′- CTCGCTTCGGCAGCACAU6-3′
		Antisense:5′-AACGCTTCACGAATTTGCGT-3′
